# Diagnosis, Pathogenesis and Treatment of Muscular Dystrophy

**DOI:** 10.3390/biomedicines13081820

**Published:** 2025-07-25

**Authors:** Manuela Bozzi

**Affiliations:** 1 Dipartimento di Scienze Biotecnologiche di Base, Cliniche Intensivologiche e Perioperatorie, Università Cattolica del Sacro Cuore, Largo F. Vito 1, 00168 Roma, Italy; manuela.bozzi@unicatt.it; 2 Istituto di Scienze e Tecnologie Chimiche “Giulio Natta”—SCITEC (CNR), Largo F. Vito 1, 00168 Roma, Italy

## 1. Introduction

Muscular dystrophies are a group of inherited genetic disorders that involve an ever-growing number of genes. Nowadays, more than 50 genes have been linked to different muscular dystrophies [1]. This genetic variability accounts for the wide heterogeneity of phenotypes observed in patients. From a clinical and pathological standpoint, muscular dystrophies are characterized by varying ages of onset and a broad spectrum of symptoms, including severe forms that impact other organs and tissues, particularly the central nervous system. Nevertheless, muscular dystrophies exhibit common histopathological and molecular characteristics that reflect disease progression. The primary shared feature is the accumulation of fibrotic tissue, which results from muscle fiber degeneration and cell death, ongoing inflammation, and increased oxidative stress. These factors contribute to the infiltration of inflammatory cells and the oxidation of lipids and proteins [2]. In the most severe forms of muscular dystrophy, cardiomyopathy and respiratory dysfunction occur during disease progression, severely affecting the patients’ quality of life and strongly reducing their lifespan. Duchenne muscular dystrophy, Myotonic dystrophy type 1, and Facioscapulohumeral muscular dystrophy are some of the most common forms of muscular dystrophies. In particular, due to mutations in the dystrophin gene, Duchenne muscular dystrophy has an incidence of about 1 in 5000 live males every year [3].

Early diagnosis is essential for effectively managing disease progression, and it should include genetic testing. However, establishing an accurate diagnosis can be challenging, as individuals with mutations in the same gene may present with diverse clinical manifestations. Therefore, a comprehensive approach integrating clinical evaluation, molecular testing, and tissue analysis is critical for achieving a precise diagnosis and informing a reliable prognosis [4,5].

At present, there is no definitive cure for muscular dystrophy. Glucocorticoid therapy remains the cornerstone of muscular dystrophy treatment, helping to delay disease progression and improve muscle strength. However, its use is associated with significant side effects, including adrenal suppression, growth delay, weakened bone health, and the onset of metabolic syndrome [6]. This highlights the need to develop innovative therapeutic strategies.

Therapies based on various molecular mechanisms are progressively entering clinical use for muscular dystrophy. For instance, antisense oligonucleotides designed to modify the splicing process of dystrophin have been developed to bypass out-of-frame dystrophin mutations [7]. Several of these treatments, including eteplirsen [8], golodirsen [9], viltolarsen [10], and casimersen [11], have received approval from the U.S. Food and Drug Administration. However, while these drugs demonstrate a broad safety profile, their effectiveness remains limited [7].

Significant efforts have been dedicated to developing gene transfer-based therapies using adeno-associated virus vectors. Nevertheless, this approach has yielded limited benefits to date due to several key challenges, which include the restricted capacity of adeno-associated viruses to package large exogenous DNA constructs, difficulties in effectively delivering the healthy gene to all skeletal muscles, including the cardiac muscle, and the immune response triggered both by the adeno-associated virus vector itself and the protein product of the transferred gene [12].

This Editorial refers to the Special Issue “*Diagnosis, Pathogenesis and Treatment of Muscular Dystrophy*”. This Special Issue highlights the multifactorial nature of muscular dystrophies, addressing the various aspects related to them. Some articles focus on precise diagnostic procedures, some on managing the different complications that arise during disease progression, while others explore the effects of specific therapeutic protocols. The contributions of this Special Issue are briefly described in the next paragraph.

**An Overview of the Published Articles**

1. De Paepe reviewed recent research on the therapeutic potential of amino acid and derivative supplements in the treatment of Duchenne muscular dystrophy. Studies have shown altered levels of circulating amino acids in both patients with Duchenne muscular dystrophy and animal models compared to healthy controls, highlighting the need for amino acid supplementation to address these imbalances. The effects of such supplementation, as observed in both *mdx* mice (a commonly used Duchenne muscular dystrophy model) and human patients, are thoroughly documented. The review underscores the pressing need for further clinical trials to validate these findings in human populations.
2. The progression of Duchenne muscular dystrophy is characterized by the gradual decline of both respiratory and cardiac function, ultimately leading to premature death. In their review, Novelli and colleagues reported that the majority of Duchenne muscular dystrophy patients develop dilated cardiomyopathy. The genotyping of patients with primary dilated cardiomyopathy has identified over 20 causative genes, including certain dystrophin variants that do not result in skeletal muscle involvement. In Duchenne muscular dystrophy, mutations associated with more severe cardiac complications are localized to specific exons of the dystrophin gene. Notably, many patients with Duchenne muscular dystrophy who experience significant cardiac dysfunction also carry pathological variants in genes unrelated to dystrophin. To enhance prognosis and develop personalized treatment strategies, Novelli and colleagues emphasized the need to perform complete genotyping of the patient, utilizing next-generation sequencing technology. However, for an accurate diagnosis, genotyping must be integrated with conventional diagnostic tools, including echocardiography and cardiac magnetic resonance imaging.
3. Neuhoff and colleagues conducted a genetic screening of 403 male patients affected by dystrophinopathy. This analysis led to the identification of 13 previously unreported dystrophin gene variants. The location of these mutations within the dystrophin gene provided insights into the likely severity of disease progression. By correlating the genetic test with clinical symptoms and muscle biopsy findings, the authors established genotype–phenotype relationships. They concluded that genetic testing alone is insufficient for accurate prognosis; instead, a comprehensive approach combining clinical evaluation, molecular analysis, and histological examination is essential to reliably predict disease severity.
4. In their article, Yamamoto and colleagues explored the relationship between the fragmented QRS complex (fQRS) and cardiac dysfunction in patients with Duchenne muscular dystrophy. The study examined 184 patients under 20 years old using an 18-lead synthesized electrocardiogram. fQRS, which is associated with myocardial fibrosis, was detected in 91% of the patients, with higher prevalence in older individuals and those experiencing cardiac dysfunction. The findings revealed that fQRS in the lateral leads correlates with cardiac dysfunction and left ventricular dilation, while fQRS in the anterior leads is linked to age. Myocardial fibrosis, which initially affects the lateral walls, is considered a precursor to cardiac dysfunction. fQRS may serve as a simple and effective indicator for monitoring myocardial damage in patients with Duchenne muscular dystrophy.
5. Siemionow and colleagues developed a novel cell therapy for Duchenne muscular dystrophy using human dystrophin-expressing chimeric (DEC) cells. These cells were created by fusing myoblasts derived from both healthy donors and individuals affected by Duchenne muscular dystrophy. In their first article, the authors present the results of an in vitro analysis of DEC cells. The findings revealed a high level of chimerism and robust expression of key muscle proteins, including dystrophin, desmin, and a myosin-heavy chain. Furthermore, the DEC cells exhibited healthy mitochondria (transferred from the healthy donor), as well as chimeric mitochondria.
6. In their second article, Siemionow and colleagues investigated the effects of administering human dystrophin-expressing chimeric cells to immunodeficient *mdx* mice via the systemic-intraosseous route. The authors examined the histological and morphological characteristics of the cardiac, diaphragm, and gastrocnemius muscles. Their analysis revealed a shift in muscle fiber size distribution toward a wild-type phenotype, along with a significant increase in the mean Feret’s diameter compared to vehicle-injected controls. These therapeutic effects were dose-dependent and persisted for up to 180 days.
7. Myotonic dystrophy type 1 is a rare inherited neuromuscular disorder due to an autosomal-dominant trinucleotide cytosine–thymine–guanine (CTG) repeat expansion in the noncoding part of the myotonic dystrophy protein kinase (*DPMK*) gene. Clinical symptoms consist of muscle weakness and myotonia involving many other organs. Respiratory and cardiac dysfunctions mainly contribute to a reduced lifespan. Basa and colleagues carried out a study aimed at identifying the main risk factors for developing sleep-disordered breathing. Their findings show that conventional predictive tools like pulmonary function tests and symptom questionnaires often fail to detect sleep-disordered breathing in children with Myotonic dystrophy type 1 due to factors such as intellectual disability and disease-specific variability. Notably, no clear correlation was found between genotype and sleep-disordered breathing severity, and central nervous system abnormalities may play a contributing role. The study underscores the need for alternative diagnostic tools, such as forced oscillometry and endoscopic airway assessments. It also calls for the development of more sensitive capnometry standards and long-term studies to better understand respiratory impairment progression. The study reinforces the importance of tailored early respiratory care and the potential utility of simplified monitoring tools like overnight oximetry in managing sleep-disordered breathing in children with Myotonic dystrophy type 1.
8. In their study, Cho and colleagues assessed the effectiveness of airstacking techniques using an affordable compact manometer that provides digital pressure feedback. Their findings showed no significant improvement in patients’ respiratory functions. This outcome was attributed to the fact that participants had already been practicing airstacking prior to the study, likely diminishing the added benefit of the digital feedback device. Nevertheless, the authors noted that such digital tools can assist caregivers by optimizing insufflation pressure and reducing their musculoskeletal pain.
9. Sheptulina and colleagues examined the ultrasound characteristics of the rectus femoris muscle in patients affected by metabolic dysfunction-associated steatotic liver disease and explored how these features relate to body composition, muscle strength, and bone mineral density. The authors found that higher echogenicity was positively associated with body fat percentage and negatively with muscle mass and strength. Greater subcutaneous fat thickness was linked to reduced muscle mass and strength. A larger anterior–posterior diameter of the rectus femoris muscle correlated positively with muscle mass, strength, and lumbar spine bone mineral density, while muscle stiffness was inversely related to body fat percentage and positively associated with muscle mass and lumbar bone mineral density. The authors concluded that ultrasound is a reliable tool to assess muscle quality and quantity in patients with metabolic dysfunction-associated steatotic liver disease. Their findings significantly correlate with body composition, strength, and bone mineral density, suggesting its usefulness in the early detection of musculoskeletal complications and osteoporosis risk.
10. Facioscapulohumeral muscular dystrophy is a hereditary muscle disorder with an incidence of approximately 1 in 15,000 to 20,000 individuals. In patients with Facioscapulohumeral muscular dystrophy, the double homeobox 4 (*DUX4*) gene, which is normally silenced in healthy adults, is stochastically misexpressed in skeletal muscle. Facioscapulohumeral muscular dystrophy progresses gradually and affects skeletal muscles asymmetrically, leading to progressive mobility impairments. In their study, Kakimoto and colleagues investigated the effects of a novel gapmer antisense oligonucleotide, *MT-DUX4-ASO*, in transgenic mice expressing the *DUX4* gene, without the use of a ligand for drug delivery. The authors demonstrated that the subcutaneous administration of 10 mg/kg of *MT-DUX4-ASO* every other week effectively suppressed *DUX4* expression, leading to restored muscle histology and improved motor functions.

## 2. Conclusions

Although there is currently no definitive cure for muscular dystrophies, significant progress has been made in recent years in developing therapeutic protocols. Three articles published within this Special Issue present promising treatment strategies for combating Duchenne muscular dystrophy (Contributions 5 and 6) and Facioscapulohumeral muscular dystrophy (Contribution 10).

While a definitive cure remains elusive, the advancement of diagnostic strategies is essential for the effective management of disease progression. Several articles in this Special Issue propose protocols for the early detection and monitoring of cardiomyopathy (Contributions 2, 3, and 4), respiratory dysfunction (Contribution 7), and muscle strength decline (Contribution 9). Additionally, an innovative technique for managing respiratory dysfunction is explored (Contribution 8), and the therapeutic potential of amino acid supplementation is reviewed (Contribution 1).
